# Distribution of vesicle pools in cerebellar parallel fibre terminals after depression of ectopic transmission

**DOI:** 10.1371/journal.pone.0200937

**Published:** 2018-07-19

**Authors:** Katharine L. Dobson, Zoe H. Smith, Tomas C. Bellamy

**Affiliations:** School of Life Sciences, University of Nottingham Medical School, Nottingham, United Kingdom; Aix-Marseille Universite, FRANCE

## Abstract

At parallel fibre terminals in the cerebellar cortex, glutamate released outside of the active zone can activate AMPA receptors on juxtaposed Bergmann glial cell processes. This process is termed “ectopic” release, and allows for directed transmission to astroglial cells that is functionally independent of synaptic transmission to postsynaptic Purkinje neurons. The location of ectopic sites in presynaptic terminals is uncertain. Functional evidence suggests that stimulation of parallel fibres at 1 Hz exhausts ectopic transmission due to a failure to rapidly recycle vesicles to the ectopic pool, and so would predict a loss of vesicles in the near vicinity of extrasynaptic glial processes. In this study we used this stimulation protocol to investigate whether the distribution of vesicles within the presynaptic terminal is altered after suppression of ectopic release. Stimulation at 1 Hz had only a minor impact on the distribution of vesicles in presynaptic terminals when analysed with electron microscopy. Vesicle number and terminal size were unaffected by 1 Hz stimulation, but the relative abundance of vesicles in close proximity to the active zone was marginally reduced. In contrast, the fraction of vesicles facing glial membranes was unchanged after suppression of ectopic transmission. 1 Hz stimulation also resulted in a small but statistically-significant increase in the distance between glial membrane and presynaptic terminal, suggesting withdrawal of glial membranes from synapses is detectable in ultrastructural anatomy within minutes. These results raise doubts about the location of ectopic release sites, but indicate that neuron-glial association varies on a dynamic time scale.

## Introduction

The synapses of the cerebellar cortex are enveloped by processes of a unique type of astrocyte, the Bergmann glial cell [1,2]. Close association of Bergmann glia and the synapses onto the principal neuron of the cortex, the Purkinje neuron, is essential for maintenance of normal synaptic transmission [3,4] and regulation of synaptogenesis [5]. Bergmann glia express a Ca^2+^-permeable form of AMPA receptor that is activated during synaptic transmission [6–8]. Genetic deletion or alteration of the subunit composition of Bergmann glial AMPA receptors leads to dramatic changes in cortical structure and function: Bergmann glial processes withdraw from synapses, synaptic glutamate transients are prolonged, synaptic connectivity is remodelled, and motor coordination is compromised [3–5].

A series of studies have determined that transmission from presynaptic parallel and climbing fibre terminals to the extrasynaptic Bergmann glia is functionally independent of transmission to the postsynaptic Purkinje neurons. Unitary events are desynchronized [9], the magnitude of short-term plasticity differs, the calcium channel isoforms linked to release onto the two cells are pharmacologically distinct [10], and presynaptic long-term plasticity mechanisms are markedly different [11–13]. This functional independence has been explained by the occurrence of ectopic transmission–release of glutamate from presynaptic terminals at sites outside of the active zone, which provides a privileged route for neuron-glial transmission. It has been hypothesized that this ectopic transmission allows glial processes to locate and surround synapses, and so the loss of glial AMPA receptors that are poised to receive ectopic input results in the withdrawal of glial processes from presynaptic terminals that causes dysfunction in cerebellar processing [3].

Despite this wealth of functional evidence for independent routes for transmission to neurons and glia, the location of ectopic release sites remains uncertain. Matsui et al. [14] reported the presence of vesicles in presynaptic terminals of parallel fibres that were positioned close to the plasma membrane at sites juxtaposed to glial processes that contained AMPA receptor subunits. The authors hypothesised that these vesicles represented the ectopic pool. We have previously described a form of input-specific, long-term depression (LTD) of transmission to Bergmann glia caused by repetitive stimulation of parallel and climbing fibres in the 0.1–1 Hz range [11,15]. The mechanism of this LTD appears to be a failure to recycle vesicles to the ectopic pool, leading to progressive depletion of release-ready vesicles at ectopic sites [13].

Collectively, these reports suggest that structural rearrangements of the presynaptic terminal and glial sheath, and redistribution of presynaptic vesicle pools, may accompany changes in the strength of ectopic transmission to Bergmann glia.

In this study, we stimulated parallel fibres at different frequencies, to investigate how the ultrastructure of the parallel fibres synapses are affected by activity-dependent depression of ectopic transmission. We recorded from individual Bergmann glia with the whole-cell patch clamp technique, using Lucifer yellow to guide identification of stimulation sites in cerebellar slices post-fixation with electron microscopy, and so determine the abundance and distribution of presynaptic vesicles and extrasynaptic glial processes at synapses that had been stimulated at 0.033 Hz (which does not depress ectopic transmission), and 1 Hz (which does depress ectopic transmission). By comparison of synapses stimulated in these ways, we tested the prediction that depression of ectopic transmission would be associated with a loss of vesicles positioned at sites opposite extrasynaptic glial processes. We also examined whether depression of ectopic transmission was associated with a change in the coverage of synapses by glial processes.

The results showed small but detectable changes in the fraction of vesicles located within close proximity of the active zone and an increase in the distance between terminal and glial envelope, in synapses stimulated at 1 Hz compared to 0.033 Hz. In contrast, there was no detectable difference in the fraction of vesicles juxtaposed opposite glial membranes, casting doubt of the identity of such vesicles as the hypothesised ectopic pool.

## Materials and methods

### Ethical statement

Rats (age 16–20 days) were humanely killed by cervical dislocation. All experiments were performed according to policies on the care and use of laboratory animals of British Home Office and European Community laws. The University of Nottingham Animal Welfare and Ethical Review Body approved the experiments. All efforts were made to minimize animal suffering and reduce the number of animals used.

### Cerebellar slice preparation

Transverse cerebellar slices (300 μm) were prepared from 16- to 20-day old Wistar rats of either sex. Animals were humanely killed by cervical dislocation, decapitated, and the cerebellum rapidly excised and sliced using a vibrating microtome (Leica VT1000S) in chilled, sucrose-based artificial cerebrospinal fluid (aCSF) containing (mM): sucrose (206), KCl (2.8), CaCl_2_ (1), MgCl_2_ (1), MgSO_4_ (2), NaHCO_3_ (26), glucose (10), ascorbic acid (0.4), and NaH_2_PO_4_ (1.25). Slices were allowed to recover for 1 hour at 32°C, with a further 30 min at room temperature, in aCSF containing (mM): NaCl (126), KCl (3), NaH_2_PO_4_ (1.2), NaHCO_3_ (25), glucose (15), MgSO_4_ (2), and CaCl_2_ (2). For recording, slices were transferred to an immersion chamber and perfused with aCSF containing a lower concentration of MgSO_4_ (1 mM). All solutions were continuously bubbled with carbogen (95% O2, 5% CO_2_) throughout.

### Electrophysiology

Borosilicate recording electrodes were manufactured as previously described [15]. Internal solution consisted of (mM): K-gluconate (110), KCl (5), HEPES (50), EGTA (0.05), MgSO_4_ (4), ATP (4), GTP (0.2), phosphocreatine (9), and pH to 7.4 with 1 M KOH, plus 1 mg/mL Lucifer yellow. Whole-cell voltage clamp recordings were made from Bergmann glia (holding potential -80 mV) somata in the Purkinje cell layer. Currents were low pass filtered at 4–5 kHz and sampled at 25 kHz, using Spike2 software (CED, Cambridge, UK). Series resistances ranged from 5 to 15 MΩ and were uncompensated.

Parallel fibres were stimulated with a patch electrode (~1–2 MΩ) filled with bath solution and positioned in the midpoint of the molecular layer, connected to an isolated constant current stimulator (100 μA, 80 μs; Digitimer, Welwyn Garden City, UK). Stimulus was delivered as a pair of pulses with a 100 ms interval at a frequency of either 0.033 Hz or 1 Hz; a stimulus protocol that has been shown to be the most effective mechanism for depression of ectopic release [15], due to the increased release probability for the facilitated second pulse.

### Recording protocol and fixation

Samples were processed according to a protocol modified from Marra et al. [16]. Following identification of a suitable glial cell and positioning the stimulating electrode, a brightfield image (4X) was captured to enable realignment of the slice during later steps. Cells were then patched with the whole-cell recording configuration and were loaded for 10 minutes to ensure that fine structures were infiltrated with dye. Cells were randomly assigned to one of three stimulation protocols (no stimulation, 0.033 Hz, or 1 Hz), each of which had a duration of 10 minutes, giving a total duration of 20 minutes recording. The micropipette was then slowly withdrawn to reseal the cell and minimise dye leakage during subsequent steps.

Slices were rapidly fixed in a calibrated microwave (700 W, 8 s) in a fixative solution containing 6% paraformaldehyde and 2% glutaraldehyde prepared in PBS. Slices were then blocked for 1 hour in 100 mM glycine solution, rinsed for 1 minute in 100 mM NH_4_Cl, and washed three times in PBS. Slices were then returned to the recording chamber (prefilled with PBS) and orientated to match the image acquired at the outset of the protocol.

**Fig 1:**
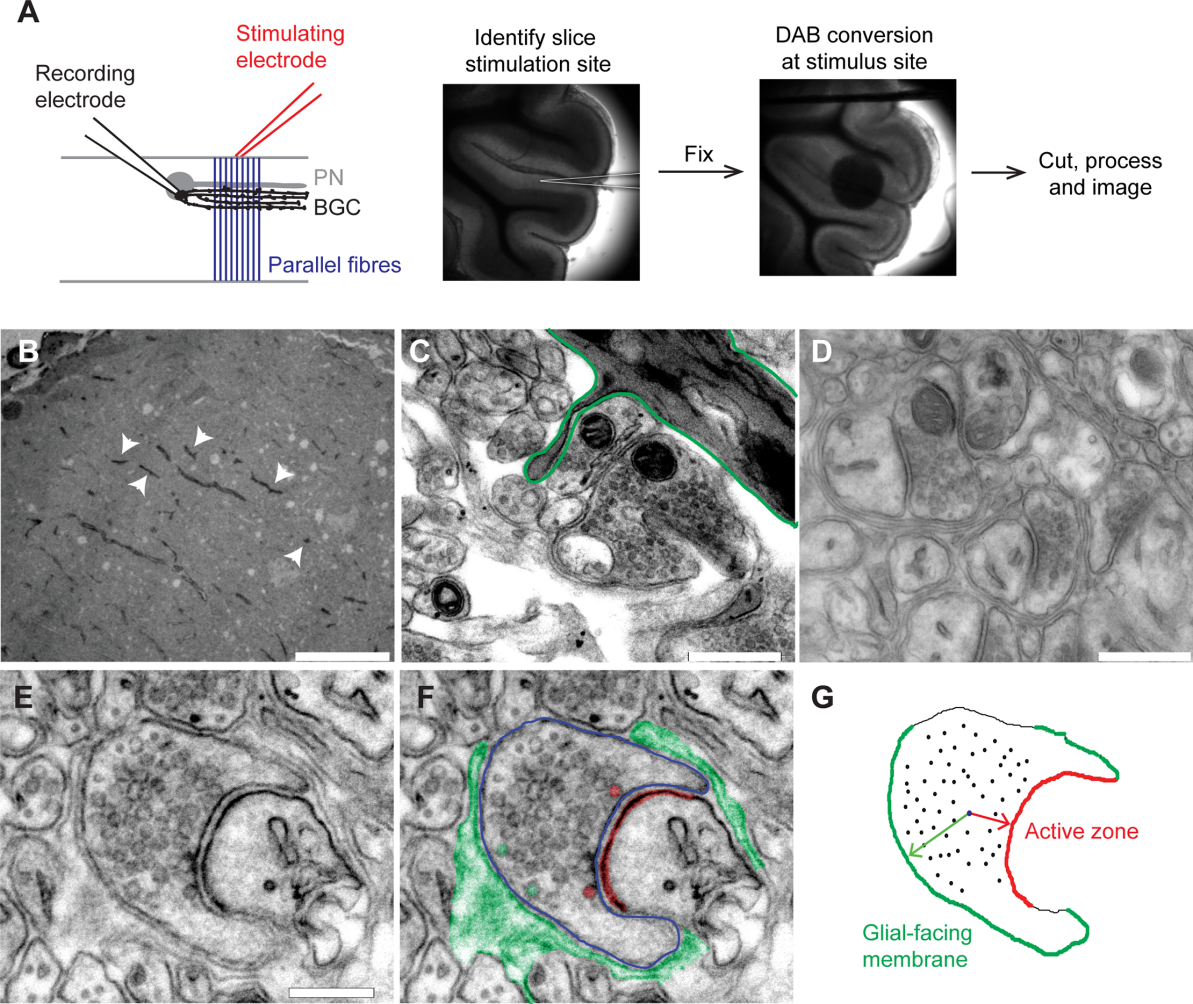
Analysis of vesicle distribution in presynaptic terminals. **A)** Illustration of recording and stimulation configuration. A Bergmann glial cell (black) was recorded with whole-cell patch clamp technique, with an internal solution containing 1 mg/ml Lucifer yellow. A stimulating electrode was placed in the centre of the molecular layer to activate parallel fibres (blue). **B)** After DAB treatment and photoconversion, electron-dense fibres in the molecular layer (arrow heads) confirm the recording and stimulation site. Scale bar = 25 μm. **C)** Bergmann fibres with electron dense deposits can be observed at higher power (green outline) and used to orient analysis to the stimulated region of the molecular layer. Scale bar = 500 nm **D)** Individual presynaptic terminals can be identified within the stimulated region. Scale bar = 500 nm. **E)** Individual terminals were isolated, contrast-adjusted and used to assess vesicle distribution. Scale bar = 150 nm. **F)** Manual labelling of presynaptic terminal (blue line), extrasynaptic glial processes (green fill) and postsynaptic density (red fill) was carried out for each terminal. In this case, vesicles located close to the active zone (red fill) or glial facing membrane (green fill) were visible. **G)** After manual labelling of structures, the centre of each visible vesicle was marked as a point. Export of the coordinates from imaging software allowed reconstruction of the terminal in Matlab. For every vesicle (black dots), the shortest distance to the nearest glial facing membrane (green) and active zone (red) was calculated.

PBS was replaced with 1 mg/mL 3,3'-diaminobenzidine (DAB) solution prepared in oxygenated PBS and incubated in the dark for 10 minutes. This was replaced with fresh oxygenated DAB solution and the photoconversion reaction initiated by illumination with high intensity blue light through a 40X objective lens (Nikon 100 W super high pressure mercury lamp; CFP filter set: Ex 434 ± 8.5 nm, Em 479 ± 20 nm). The DAB solution was bubbled with carbogen throughout illumination via a fine bore capillary tube, and the conversion reaction time ranged from 10 to 45 minutes. Conversion reactions were monitored every 5–10 minutes and terminated when a defined dark region was visible on the slice (Fig 1A). Slices were then washed firstly with PBS (3 × 10 minutes), and secondly with 0.1 M sodium cacodylate buffer (CB; 3 × 10 minutes). Slices were then stored at 4°C prior to further processing.

Peak identification in frequency histograms was carried out in Matlab by curve fitting using a kernel distribution function with bandwidth determined using Silverman’s rule. Area under the fitted curve was estimated using a trapezoidal numerical integration at the location determined using the ‘findpeaks’ function.

### Slice processing for electron microscopy

Slices were incubated for 1 hour in 1.5% potassium ferrocyanide/1% osmium tetroxide (wt/vol) in CB, washed with CB (5 × 10 minutes), and incubated for 1 hour in 1% osmium tetroxide in CB.

Slices were then washed again with CB (3 × 10 minutes) before a 10 minute incubation in 50% ethanol. Samples were then stained *en bloc* for 1 hour in 4% uranyl acetate in 70% ethanol before being dehydrated in rising concentrations of ethanol (2 × 75%, 2 × 90% and 2 × 100%, 5 minutes each). Slices were then further dehydrated by overnight incubation in a 1:1 solution of propylene oxide and resin (5 mL araldite CY212 resin, 3 mL agar 100 resin, 11 mL DDSA, 0.4 mL dibutyl phthalate and 0.3 mL DMP 30). Resin infiltration was performed by transferring the samples into freshly prepared resin for a period of 12 hours after which the resin was changed and incubated for a further 12 hours. Samples were then flat embedded between two sheets of ACLAR® film (Agar Scientific, Essex, UK) and cured for 48 hours at 60°C. The embedded sample was then affixed to a resin blank and sectioned from the photoconverted face using an ultramicrotome (70 nm thickness) and embedded in a copper mesh grid.

### Electron microscopy and data analysis

Ultrathin samples were imaged by transmission electron microscopy (FEI Tecnai 12 Biotwin TEM, 120kV, magnification up to 43,000X) using the previous slice images to assist with locating the loaded cell in each slice. Ten high-power images of synapses located within the arborisation of the loaded Bergmann glial cell were captured for each ultrathin section, and analysed by researchers blinded to the stimulation paradigm employed for each sample.

EM images were analysed in Image J (available at http://rsb.info.nih.gov/ij; Wayne Rasband, National Institutes of Health, Bethesda, MD). Freehand tools were used to trace the perimeter of each presynaptic terminal, the postsynaptic density, and extrasynaptic glial membranes that abutted the terminal (Fig 1F). The centre of each vesicle within the terminal was then marked as a point. The coordinates of each of these anatomical features were then exported to Matlab (The MathWorks, Inc., Natick, Massachusetts, US), where a bespoke script was used for further analysis (S1 File). The terminal membrane was classified into active zone and glial-facing membrane regions by demarcating those sections directly facing postsynaptic density and extrasynaptic glial processes, respectively. Finally, for every vesicle, the shortest distance (measured as the Euclidean distance) to the nearest active zone or glial-facing segment was calculated (Fig 1G). This procedure returned the key parameters for analysis: the distribution of distances for vesicles from active zone and glial facing regions, and the fractional coverage of the terminal by glial processes. We also quantified the total number of vesicles and size of terminal for all conditions.

### Statistical analysis

For comparison of anatomical parameters between stimulation conditions, we used a Kruskal-Wallis ANOVA test with Dunn’s correction for multiple comparisons. For hypothesis testing, we used contingency tests (Fisher’s exact test) to compare the number of vesicles within <50 nm of active zone and glial facing membrane in terminals stimulated at 0.033 Hz and 1 Hz. Aggregate data are the mean ± s.e.m. of multiple cells as indicated in figure legends.

## Results

To examine synapse anatomy before and after depression of ectopic transmission, we adopted the following strategy (Fig 1A): a stimulating electrode was positioned in the middle of the molecular layer, on the surface of an acutely-isolated, sagittal cerebellar slice. A Bergmann glial cell located approximately 20–30 μm into the neuropil was identified, and a whole-cell recording established, using an internal solution that contained 1 mg/ml Lucifer yellow. Paired pulse stimulation of the molecular layer was carried out for 10 min at 0.033 Hz or 1 Hz, with a high stimulation intensity (100 μA, 80 μs). Slices were rapidly fixed, returned to the recording chamber, and then incubated with DAB. The stimulated region was then exposed to intense blue light to trigger photoconversion of DAB to an electron-dense product (Fig 1A; see Methods for full details). The labelled region was trimmed with a biopsy punch, and then processed for EM imaging. This protocol resulted in electron-dense labelling of the recorded Bergmann glial cell, which when imaged with the electron microscope could be identified as dark processes ramifying through the neuropil (Fig 1B and 1C). This acted as a “signpost” to orientate the search for synapses to the region of the molecular layer from which electrophysiological data had been obtained during stimulation of parallel fibres.

Synapses within this stimulated region were identified manually, and images taken at 43,000X magnification (Fig 1E). Consistent with the report by Matsui et al. [14], we were able to identify multiple presynaptic terminals in which vesicles appeared to be closely located beside the plasma membrane outside of the active zone, but opposite an extrasynaptic glial process, which we took to be putative ectopic sites (Fig 1F, green).

To quantify vesicle distribution in detail, the periphery of presynaptic terminal was traced, the active zone identified as the region of the terminal directly apposed to the postsynaptic density, and the centre of each identifiable vesicle within the terminal was marked. Extrasynaptic glial process that surrounded the presynaptic terminal were also traced. This procedure yielded a map of the presynaptic terminal, with the active zone and glial-facing membranes colour coded (Fig 1G). The Euclidean distances from the centre of each vesicle to the nearest point of the active zone and glial-facing membranes were then calculated. In all, 295 synapses were analysed from 6 slices stimulated at 0.033 Hz, and 287 synapses from 7 slices stimulated at 1 Hz. We also obtained 167 synapses from 4 slices that were unstimulated, for comparison.

**Fig 2:**
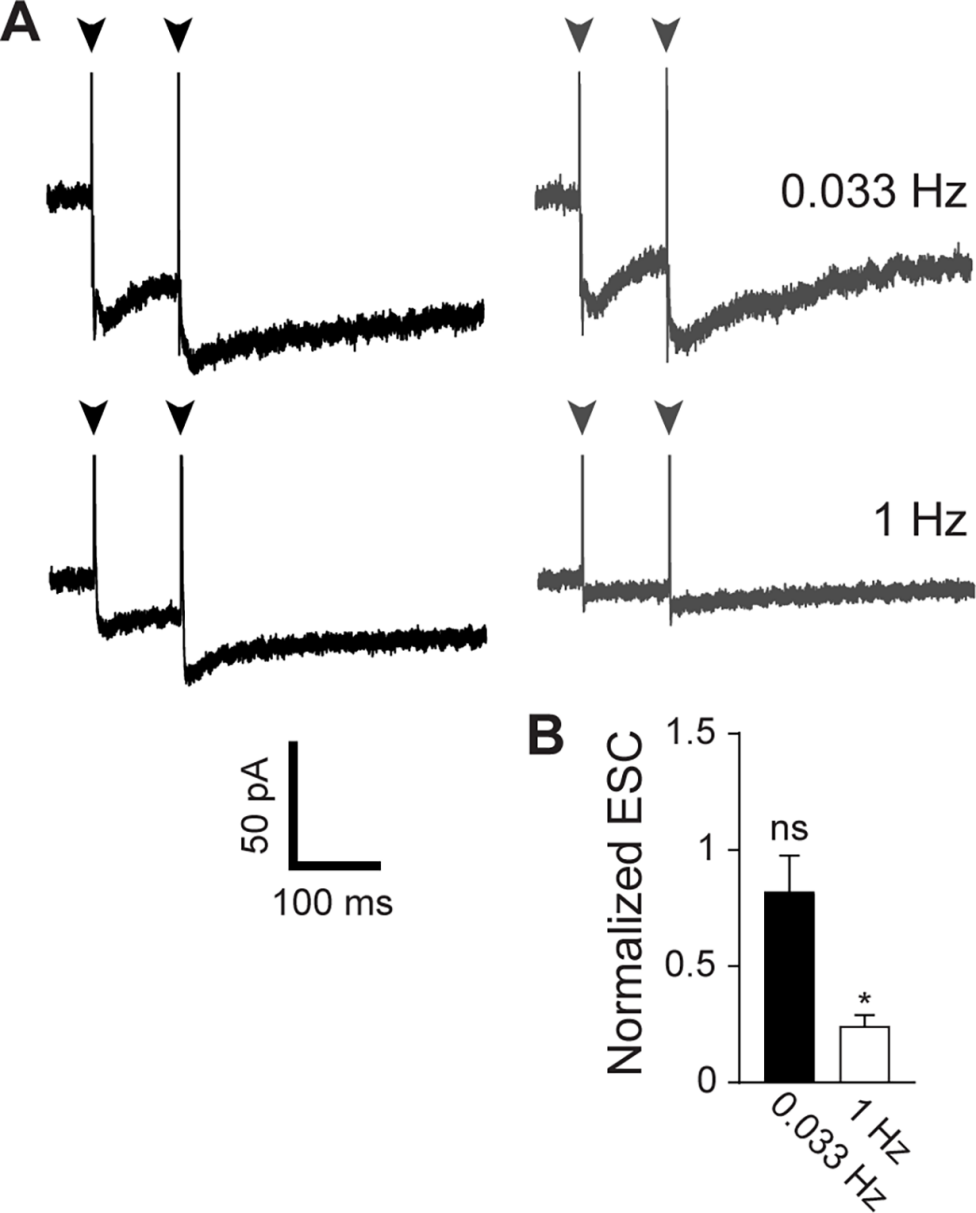
Depression of ectopic transmission. **A)** Extrasynaptic currents (ESC) recorded from Bergmann glia after paired pulse stimulation (arrows, 100 ms interval). Black traces show average of first three stimuli at 0.033 Hz (top traces) or 1 Hz (bottom traces). Grey traces show average of last three stimuli after 10 min continuous stimulation at the indicated frequencies. Stimulus artefacts have been truncated for clarity. **B**) Aggregate data (n = 9 cells for each frequency) for amplitude of extrasynaptic current after stimulation at 0.033 Hz (black) or 1 Hz (white) for 10 min, normalized to the amplitude of the first ESC. *p < 0.0001, single sample *t* test.

To confirm that our stimulation protocols successfully altered the strength of ectopic transmission, we compared the extrasynaptic currents (ESCs) [6] generated by activation of AMPA receptors and glutamate transporters in the Bergmann glia during stimulation at 0.033 Hz and 1 Hz (Fig 2A). Consistent with previous studies [13,15], 0.033 Hz stimulation for 10 min retained 82% of the initial ESC amplitude (*p* > 0.2, single sample *t* test), whereas 1 Hz stimulation depressed it to 25% of initial amplitude (Fig 2A and 2B). This residual current is not glutamatergic [6].

**Fig 3:**
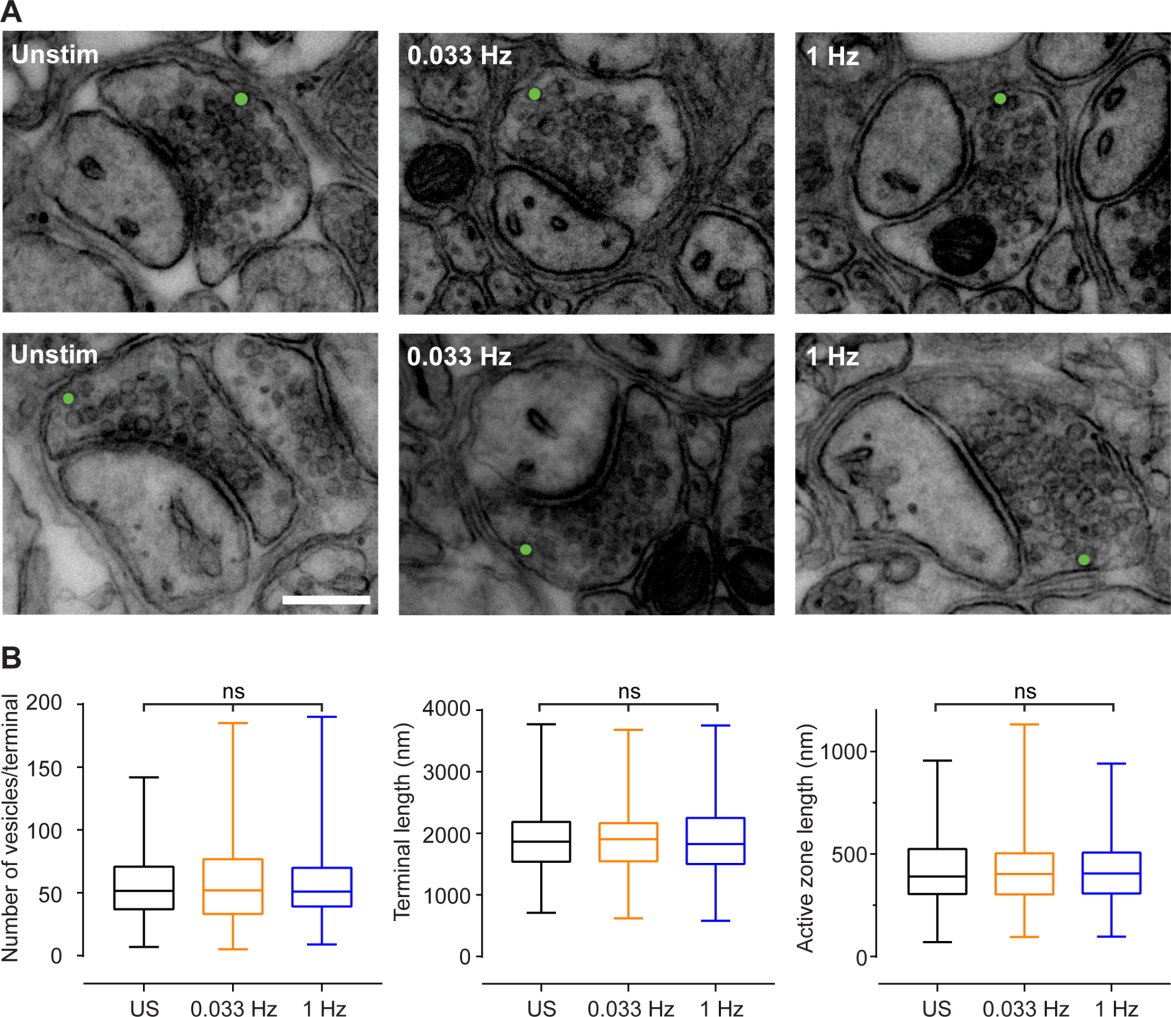
Effect of stimulation on synapse anatomy and presence of putative ectopic vesicles. **A)** Electron micrographs of synapses from each stimulation condition, with putative ectopic vesicles highlighted in green. Scale bar = 500 nm. **B)** Box and whisker (min to max) plots of the number of vesicles per terminal (left panel), terminal length (middle panel), and active zone length (right panel) for unstimulated (US, black), 0.033 Hz stimulated (orange) and 1 Hz stimulated (blue) slices. ns *p* > 0.05, Kruskal-Wallis ANOVA test with Dunn’s correction for multiple comparisons.

Stimulation at the different frequencies had no obvious impact on the gross anatomy of the terminals (Fig 3A). The average number of vesicles per terminal was not significantly different between unstimulated or stimulated terminals (at either 0.033 Hz or 1 Hz), nor was there any difference in the length of presynaptic terminal perimeter, or the length of the active zone (Fig 3B). It was also possible to visually identify putative ectopic vesicles near the glial-facing membrane in synapses from each condition (Fig 3A, individual vesicles coloured green).

**Fig 4:**
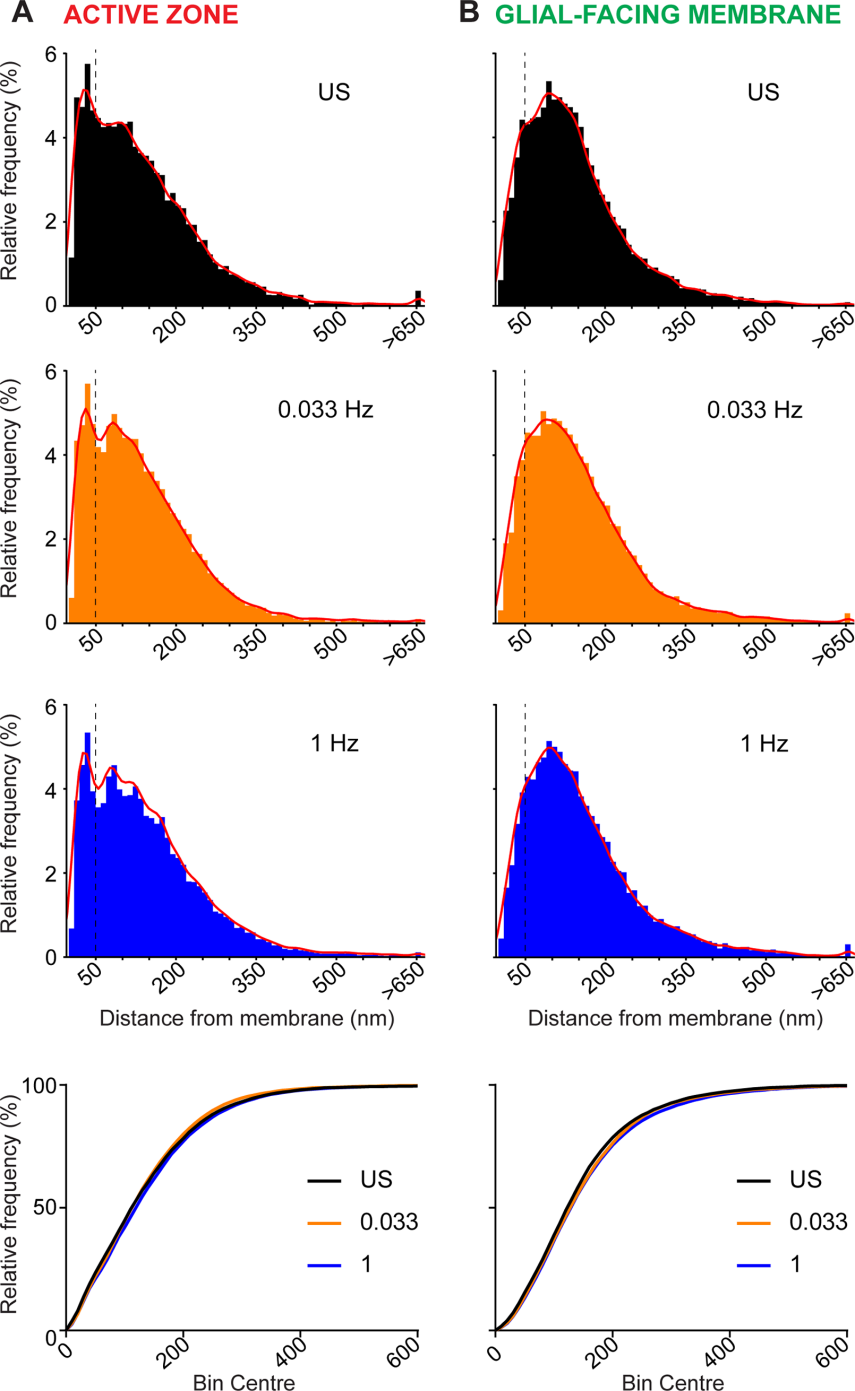
Distribution of vesicles relative to active zone and glial facing membrane. **A)** Histogram of the percentage of vesicles relative to distance from active zone (in nm), in unstimulated (black, upper panel), 0.033 Hz stimulated (orange, second panel) and 1 Hz stimulated (blue, third panel) slices. Dashed line shows separation of bimodal population at 50 nm. Cumulative frequency plot of vesicles for the three conditions are provided in the lower panel. **B)** Distribution of vesicles relative to glial facing membrane. Labels as for (A).

The distribution of vesicles throughout the terminal was analysed in detail (Fig 4). For the active zone, the distribution of distances for all vesicles appeared to have a bimodal pattern, with a narrow peak between 0 and 50 nm, and a broader peak centred at around 100 nm (Fig 4A). This is consistent with a clearly detectable population of vesicles clustered within <50 nm of the active zone membrane (estimated vesicle diameter in our population is 52.5 ± 1.39, n = 12 vesicles; 4 from each stimulation condition), which is consistent with the existence of a readily-releasable pool. The larger population therefore most likely corresponds to the reserve pool [17]. To analyse the vesicle pools in more detail, we used a kernel-fitting method to estimate the probability density function for the distribution (see Table 1, and Materials and Methods). For unstimulated synapses, and synapses stimulated at 0.033 Hz, two peaks were identified, consistent with qualitative observations, but for synapses stimulated at 1 Hz a third peak was identified at 117 nm. The probability density at the first two peaks was closely similar at the two stimulus frequencies, and peak location varied by less than the width of a bin, suggesting that the identity and relative abundance of the two principal pools did not appear to change after stimulation at either 0.033 Hz or 1 Hz (Fig 4A).

**Table 1:** Kernel distribution analysis of vesicle pools. Peak locations are stated in nm with the associated probability as a percentage determined from the probability density function values.

	*Active Zone*			*Glial facing membrane*
	*1*^*st*^ *peak nm (%)*	*2*^*nd*^ *peak nm (%)*	*3*^*rd*^ *peak nm (%)*	*1st peak nm* *(%)*
**US**	32 *(7*.*2)*	97 *(6*.*2)*	-	97 *(7*.*2)*
**0.033 Hz**	36 *(7*.*2)*	87 *(6*.*8)*	-	95 *(7*.*0)*
**1 Hz**	31 *(6*.*8)*	81 *(6*.*4)*	117 *(5*.*9)*	97 *(7*.*1)*

In contrast, the distribution of vesicles relative to the glial-facing membrane appeared to be a single population with a skewed Gaussian distribution (Fig 4B). Vesicles were present within <50 nm of the glial-facing membrane, but there did not seem to be a discernible subpopulation analogous to the readily releasable pool at the active zone. Stimulation of parallel fibres at 0.033 Hz and 1 Hz did not result in overt changes in the shape of the distribution of vesicles, the peak distance, or probability density at the peak, relative to extrasynaptic glial processes (Fig 4B).

To explicitly test the central prediction of our hypothesis–that glial-facing vesicles are the ectopic pool and that stimulation at 1 Hz depletes that pool–we quantified the number of vesicles within 50 nm (that is, with a midpoint within a typical vesicle diameter) of the terminal membrane at both active zone and glial facing sites (Table 2). This analysis showed a small but statistically significant decrease in the fraction of total vesicles that were within 50 nm of the active zone after 1 Hz stimulation. In contrast, there was no statistically significant difference in the abundance of these near-membrane vesicles at the glial facing membrane for terminals that had been stimulated at 0.033 Hz or 1 Hz.

**Table 2:** Contingency table of vesicle distances from presynaptic membrane. Data are expressed as number of vesicles less than 50 nm from the membrane / total number, and were analysed using two-sided Fisher’s exact tests to compare vesicle distribution between 0.033 Hz and 1 Hz stimulation.

	*Fraction <50 nm Active Zone*	*Fraction <50 nm Glial facing membrane*
**US**	0.213 (2019/9472)	0.135 (1278/9472)
**0.033 Hz**	0.200 (3479/17380)	0.117 (2033/17380)
**1 Hz**	0.192* (3107/16212)	0.113^ns^ (1836/16212)

ns *p* = 0.2890

*p = 0.0493.

Unstimulated slices appeared to have a larger fraction of vesicles within 50 nm of both the active zone and glial facing membrane following stimulation at either frequency (*p* < 0.02, Fisher’s exact tests, Table 2), consistent with increased vesicle turnover during neurotransmission [18,19].

**Fig 5:**
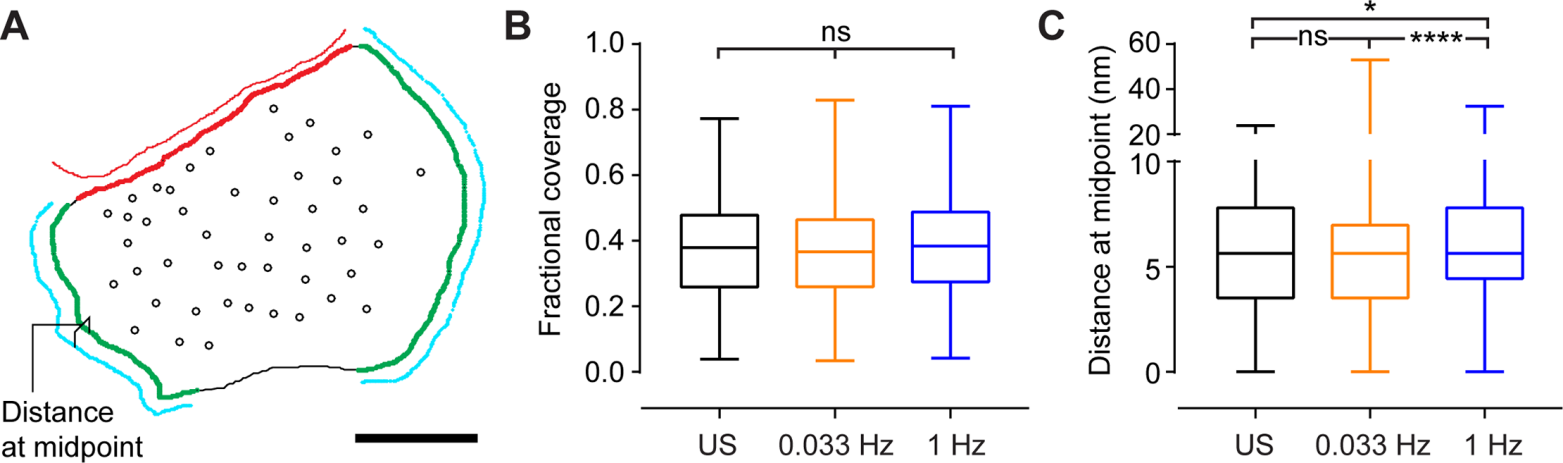
Glial coverage of presynaptic terminals. **A)** Reconstruction of the terminal from coordinates on EM image. Black line is presynaptic terminal perimeter. Red line is postsynaptic density. Cyan lines are glial process membranes. Red and green highlighting on terminal perimeter show presynaptic active zone and extrasynaptic glial-facing membrane respectively. Scale bar = 100 nm. **B)** Box and whisker (min to max) plot of fractional coverage of terminal by glial membrane (length of green highlights/terminal perimeter). **C)** Box and whisker plot of distance between glial process and glial facing membrane (cyan line and green highlight) at midpoint along glial facing membrane length. Synapses typically have 2 or 3 glial facing regions per terminal. For unstimulated slices, n = 431 glial membranes from 166 terminals; for 0.033 Hz stimulation, n = 806 glial membranes from 295 terminals; for 1 Hz stimulation, n = 801 glial membranes from 287 terminals. Statistical comparisons: ns *p* > 0.05, * *p* = 0.0232, **** *p* <0.0001 Kruskal-Wallis ANOVA test with Dunn’s correction for multiple comparisons.

Finally, we also quantified the percentage of glial coverage of the presynaptic terminals under all three conditions (Fig 5A and 5B), and measured the distance between the presynaptic terminal and the adjacent glial membrane, at the midpoint of their apposition (Fig 5C). Fractional coverage was unaffected by 1 Hz stimulation, but the mean distance at midpoint was significantly increased, from 6.01 ± 0.15 nm after 0.033 Hz stimulation, to 6.26 ± 0.10 nm after 1 Hz stimulation (unstimulated slices had a mean distance of 5.82 ± 0.14 nm). This is a modest increase in mean (and median) distance, but analysis of the distribution of values (Fig 5C) indicates a shift from shorter to greater distances predominating in the 1 Hz population.

## Discussion

Ectopic release is a recently defined phenomenon, characterised as release of neurotransmitter through vesicular exocytosis in regions of the presynaptic terminal other than the active zone [20,21]. Ectopic release has been described at several classes of synapses, including cerebellar parallel and climbing fibre inputs to the principal output neuron of the cerebellar cortex, the Purkinje neuron [9,20]. The anatomical basis and physiological roles of ectopic transmission in the cerebellar cortex remain poorly understood, however. Here, we attempted to link the abundant functional evidence for independent transmission from presynaptic terminals to postsynaptic neurons and extrasynaptic glia [10,12,13], to anatomical changes in the distribution of vesicles within the terminal. Instead, we found that stimulation conditions that successfully depressed ectopic transmission had no obvious impact on the number of vesicles within the terminals, or the spatial distribution of the vesicles relative to the glial-facing membrane.

Analysis of the distribution of vesicles relative to the active zone showed a peak centred around 30–40 nm from the presynaptic membrane, consistent with the presence of a readily-releasable pool. Stimulation of the synapses at 0.033 Hz and 1 Hz had no detectable impact on the relative size of this pool, but led to minor redistribution of vesicles within smaller second and third peaks at greater distances from the active zone (Table 1; Fig 4). These rearrangements likely reflect turnover of vesicles in recycling and reserve pools, as a consequence of the increased rate of presynaptic release.

In contrast, the distribution of vesicles relative to regions of the terminal facing glial membranes showed a single broad peak centred around 100 nm (Table 1; Fig 4). Stimulation at either frequency had no detectable impact on the spatial organisation of vesicles relative to these putative ectopic release sites. Crucially, a direct test of the hypothesis that the number of vesicles located within 50 nm of the glial facing membrane would decrease due to depletion of the putative ectopic pool, showed no statistically-significant change in relative abundance.

This inconsistency between functional and anatomical data can be explained by a number of hypotheses. First, it is possible that experimental limitations led to misidentification of stimulated synapses. Although the identification of synapses was directed by labelling the recorded Bergmann glial cell, it is possible that some synapses lying within the arborisation of the glial cell were not stimulated to release ectopic vesicles, and so were inappropriately counted within the 1 Hz population. Arguing against this possibility is the fact that slices were stimulated at an intensity that would recruit a large number of parallel fibres [6], that the fibres were repetitively stimulated for 10 min until no detectible ectopic transmission remained, that the search for synapses lay within a restricted region at the heart of the stimulated zone, and that statistically significant changes in vesicles at the active zone and neuron-glial interaction were detected. An estimate of the volume of neuropil stimulated under our experimental conditions can be made from data obtained in a similar preparation: rat visual cortex slices [22]. Taking the median value for excitation thresholds for axons in that study, at our stimulation intensity, parallel fire axons within a radius of 60 μm of the electrode would be activated with 100% probability. Axons up to 108 μm away would have a 50% probability of activation. With our stimulation regime, a reasonable estimate would be that axons within an area of 200 μm diameter around the stimulation site would be engaged. Given these observations, the likelihood of synapses capable of ectopic transmission evading such widespread and long-lasting stimulation seems low.

A second possible explanation is that ectopic release occurs at a very small fraction of the total number of terminals within the region of stimulation. If the ectopic pool has a very low release probability, or only a small fraction of terminals contain ectopic vesicles, then we may not be able to detect this small “signal” on a background of non-releasing terminals. This is possible, but would imply that ectopic transmission is not, as hypothesised, a feasible mechanism for guiding glial processes to ensheathe presynaptic terminals.

A third possible explanation for the lack of change in vesicle distribution is that the depression of ectopic transmission does not result from literal depletion of vesicles from the ectopic pool. Although the interpretation that ectopic depression arises from a failure to recycle vesicles to the ectopic pool rests on multiple lines of evidence [13], it is possible that instead of failure to recover and traffic vesicles to release sites, the deficit arises instead from a failure to refill emptied vesicles, or failure to initiate vesicle docking, priming and/or fusion. This would be consistent with a loss of neurotransmitter release from ectopic sites without a concomitant disappearance of vesicles from the ectopic pool.

Finally, the conflicting results could also be explained by a misidentification of the ectopic release sites. Although the anatomical evidence for docking of vesicles in the terminal at sites juxtaposed to glial membranes is suggestive, it may be that ectopic sites depleted by repetitive stimulation lie elsewhere. The failure to link functional and anatomical evidence suggests that the location of ectopic sites remains uncertain and worth revisiting.

One noteworthy additional consideration is the possibility that aldehyde fixatives may trigger vesicle release from parallel fibre terminals [23] and thus lead to alterations in presynaptic pool distribution prior to imaging. We would counter this with evidence from Rosenmund and Stevens’ investigation into the effects of glutaraldehyde fixation on glutamate release in cultured hippocampal neurons, which found that fixation lead to no detectable release [24]. Furthermore, the protocol of Marra et al [16] as followed here fixes using a combination of PFA and glutaraldehyde. This has been shown to fix tissue slices with great enough speed to preserve FM dye-loaded vesicles in various presynaptic pools, and therefore the rate of fixation is sufficient to prevent the vesicular release referred to by Smith and Reese [23]. Finally, Jung et al [25] report that at the frog neuromuscular junction that “such fixation does not have a measurable effect on the spatial relationships of docked SVs and their associated AZM macromolecules” compared to similar preparations fixed by rapid freezing when imaged via electron tomography.

Analysis of the morphology of glial processes around synapses revealed a small but statistically significant difference in the distance between the glial envelope and the presynaptic terminal. This result is consistent with the onset of the withdrawal of processes that has been reported in Bergmann glia that were genetically modified to eliminate AMPA receptor calcium influx [3], and suggests that the depression of neuron-glial transmission on the time scale of 10 min stimulation period can result in a detectable change in the ultrastructural morphology of glia on a matched time scale. Similarly dynamic changes in extrasynaptic astrocyte processes have been observed in the brainstem [26], consistent with glia being able to initiate morphological changes within minutes of a change in transmission strength.

Collectively, these observations suggest that alterations in synaptic coverage can be finely tuned to changes in the strength of ectopic transmission, but are not accompanied by overt changes in the distribution or localisation of vesicles in the presynaptic terminal. Further investigation is needed to understand how this mechanism for neuron-glial communication, which operates independently of transmission at the active zone, is localised and achieved.

## Supporting information

S1 File Matlab script for quantification of presynaptic terminal anatomical features. Length of terminal membrane, active zone, and glial facing membrane compartments are measured, along with point coordinates of all presynaptic vesicles. These values are used to calculate the Euclidean distance between the centre point of each vesicle and the nearest pixel of presynaptic terminal membrane, active zone and glial facing membrane. Fraction of total terminal length covered by glia, and midpoint distance between presynaptic terminal and glial membrane are also calculated. Script imports coordinate data from manually-labelled Image J file and exports table of distance measurements and outline image of terminal compartments and vesicles.
(M) Click here for additional data file.
