# Correction: Distribution of vesicle pools in cerebellar parallel fibre terminals after depression of ectopic transmission

**DOI:** 10.1371/journal.pone.0207642

**Published:** 2018-11-14

**Authors:** Katharine L. Dobson, Zoe H. Smith, Tomas C. Bellamy

There are errors in the Funding section. The correct funding information is as follows: This work was supported by the Biotechnology and Biological Sciences Research Council [grant number BB/J015660/1]; and the National Centre for the Replacement, Refinement & Reduction of Animals in Research [grant number NC/N003071/1] http://www.bbsrc.ac.uk/, https://www.nc3rs.org.uk/, both to TCB. The funders had no role in study design, data collection and analysis, decision to publish, or preparation of the manuscript.
